# Chloramphenicol-induced gray baby syndrome: case report and review of current literature

**DOI:** 10.3389/fped.2025.1733059

**Published:** 2026-01-12

**Authors:** Yu Fang, Rong Luo, Xiaolu Chen

**Affiliations:** 1 Department of Pediatric Neurology, Sichuan University West China Second University Hospital, Chengdu, Sichuan, China; 2 Key Laboratory of Obstetric & Gynecologic and Pediatric Diseases and Birth, Defects of Ministry of Sichuan University, Chengdu, Sichuan, China

**Keywords:** chloramphenicol, gray baby syndrome, clinical characteristics, diagnosis, treatment

## Abstract

**Background:**

Gray baby syndrome is a severe adverse reaction to chloramphenicol, with a mortality rate up to 40%.

**Methods:**

We report a case in which a 14-month-old Yi boy accidentally ingested 1.5 g of chloramphenicol (6 × 0.25 g tablets) that his grandmother had left on a coffee table, mistaking them for food, which led to and was diagnosed by circulatory failure (blood pressure: 87/50 mmHg), metabolic acidosis (pH: 7.131), hypothermia (36.3°C), serum drug level (92 μg/mL), and multiorgan damage; critical interventions included mechanical ventilation, vasopressors, and delayed continuous renal replacement therapy (initiated 18 h after ingestion). Besides his case, 19 other published cases were analyzed.

**Results:**

The child recovered fully after 3 weeks with no sequelae noted at 20-month follow-up. Literature analysis revealed 64.7% survival (11/17) and 35.3% mortality (6/17), with fatal cases consistently showing serum chloramphenicol levels exceeding 50 μg/mL.

**Conclusions:**

Despite typical mortality risks, delayed CRRT proved pivotal in reversing toxicity in our patient. Gray baby syndrome continues to occur in underserved regions, necessitating strict drug storage, serum concentration monitoring in high-risk infants, and early CRRT implementation for survival.

## 1 Introduction

Chloramphenicol is a synthetic broad-spectrum antibiotic. It was first mass-produced in the United States in 1949 and introduced into clinical practice (1). The drug is primarily metabolized in the liver, and its metabolites are excreted renally. Known adverse effects of chloramphenicol include bone marrow suppression, neuritis, toxic psychosis, hepatic injury, gray baby syndrome, myocardial toxicity, and severe cardiac dysfunction (2). Particularly in children, chloramphenicol use should be reserved for severe infections and given only when less toxic antibiotics are ineffective or contraindicated (3). Infants receiving excessive doses of chloramphenicol are at risk of fatal cardiovascular collapse, a condition known as gray baby syndrome (4–7) with a mortality rate as high as 40% (8). Although rarely reported in the past decade, gray baby syndrome persists in some resource-limited regions. Herein, we report a case of gray baby syndrome and review the literature to summarize the clinical characteristics, diagnosis, and management of this condition with the aim of facilitating timely clinical recognition and management by healthcare providers.

## 2 Case presentation

A 14-month-old boy (10 kg) of Yi ethnicity presented to the emergency department on September 22, 2023. He had accidentally ingested 1.5 g chloramphenicol (6 × 0.25 g tablets) left on the coffee table by his grandmother, which manifested as acute abdominal pain, vomiting, progressive lethargy, and coma within 3 h. At the time of admission, he showed hemodynamic instability (HR: 179 /min, BP: 87/50 mmHg, CRT: 4 s), hypothermia (36.3°C), and depressed consciousness (GCS 8). Laboratory investigations revealed severe metabolic acidosis (pH: 7.131, lactate: 10.06 mmol/L), anemia (Hb: 82 g/L), and hepato-renal impairment (ALT: 627 U/L, AST: 1,044 U/L, uric acid: 669 μmol/L), accompanied by cardiotoxicity (EF: 38%) and a critically elevated serum chloramphenicol level (92 μg/mL), confirming chloramphenicol-induced gray baby syndrome. Despite initial gastric lavage and fluid resuscitation, clinical deterioration necessitated pediatric intensive care unit admission. Aggressive management included mechanical ventilation, inotropic support (dopamine/milrinone), norepinephrine infusion, and high-dose vitamin C/B₁. Given delayed presentation (>8 h after ingestion) with established multiorgan failure, continuous renal replacement therapy (CRRT) was initiated at 18 h after ingestion for 79 h, alongside hepatoprotective agents (glutathione/bifendate). Hemodynamic stability was achieved within 4 h (EF 48%), and metabolic acidosis resolved by 10 h (lactate: 2.72 mmol/L). Key laboratory and diagnostic findings in this case are summarized in Table 1. After 3 weeks of intensive care, all laboratory and cardiac parameters normalized. The patient was discharged on October 14, 2023, with no sequelae observed during a 20-month follow-up (last assessment May 28, 2025), showing successful reversal of severe chloramphenicol toxicity through multimodal critical care intervention.

**Table 1:** Key laboratory and diagnostic findings in this case of gray baby syndrome.

Parameters		Pre-resuscitation	Post-resuscitation	Pre-CRRT	Post-CR RT	Pre-discha rge
Arterial blood gas	PH	7.131	7.353	7.232	7.489	
	BE mmol/L	−20	−13	−15.7	3	
	HCO3- mmol/L	7.2	11	10.4	26.5	
	LAC mmol/L	10.06	2.72	0.77	0.99	
Complete blood count	WBC 10^9^ /L	10.92		7.5	6.1	6.1
	Hb g/L	82		74	79	100
	PLT 10^9 ^/L	425		277	68	334
	CRP mg/L	0.54		1.3	0.8	<0.5
Liver function tests	ALT U/L	16.6		627	692	36
	AST U/L	32.9		1,044	281	43
Renal function tests	UA μmol/L	522.5		669	<30	257
	Cr μmol/L	29.3		32	11	14
Cardiac injury markers	cTnI ng/mL			0.31		<0.003
	Myo ng/mL			157.4		12.5
	CK-MB ng/mL	2.96		3.94		2.75
Cardiac findings	LVEF	38	48	62 (mild right ventricular dilatation)		
	ECG			Sinus tachycardia with T-wave abnormalities: Flattened or inverted T waves in leads II, III, aVF, V3, and V5		
Pulmonary findings	CXR	Bilateral pulmonary opacities with interstitial changes and likely areas of consolidation		Increased and indistinct bilateral pulmonary markings		
Abdominal findings	Abdominal US			Hepatomegaly with an enlarged right hepatic lobe measuring 9.3 cm in oblique diameter Ascites with a maximum depth of 3.7 cm		

pH, potential of Hydrogen; BE, base excess; LAC, lactic acid; WBC, white blood cell count; Hb, hemoglobin concentration; PLT, platelet count; CRP, C-reactive protein; ALT, alanine aminotransferase; AST, aspartate aminotransferase; UA, uric acid; Cr, creatinine; cTnI, cardiac Troponin I; Myo, myoglobin; CK-MB, creatine kinase-MB isoenzyme; LVEF, left ventricular ejection fraction; ECG, electrocardiogram; CXR, chest x-ray; US, ultrasound.

## 3 Discussion

In 1960, the Massachusetts Medical Society (4) recommended the following chloramphenicol dosing regimens: infants over 30 days old: 100 mg/kg per day; full-term neonates: 50 mg/kg per day; and preterm infants under 30 days old: 25 mg/kg per day. The risk of toxicity is relatively low when serum chloramphenicol concentrations remain within the therapeutic range of 15–25 μg/mL (2). Gray baby syndrome is associated with serum concentrations exceeding 50 μg/mL (9). The characteristic clinical presentation includes hypotonia, lethargy, ashen-gray skin discoloration, cyanosis, abdominal distension, impaired peripheral perfusion, hypotension, hypothermia, and metabolic acidosis (4–7, 10). Notably, metabolic acidosis is regarded as an early indicator of chloramphenicol toxicity (11).

Chloramphenicol overdose inhibits mitochondrial protein synthesis, which disrupts oxidative phosphorylation and causes cellular necrosis. Necrosis releases vasoactive substances, triggering microcirculatory dysfunction (12). At high doses, the drug also induces myocardial damage and dysfunction (13), thus reducing cardiac output and hepatic blood flow, further impairing drug clearance and exacerbating toxicity. Newborns, particularly preterm infants, have underdeveloped livers and relatively low glucuronidation capacity, making them highly susceptible to chloramphenicol toxicity and the highest-risk group for gray baby syndrome (7). However, with excessive chloramphenicol dosing, similar symptoms can occur even in older children and adults (11, 14–16). Although the 1960 guidelines (4) significantly reduced the incidence of chloramphenicol toxicity, cases still occasionally occur due to prescription errors or accidental ingestion.

A total of 19 cases of chloramphenicol-induced gray baby syndrome have been reported in PubMed and Wanfang Database as of May 20, 2025 (Table 2). Among them, 10 were male, 5 were female, and the sex was not mentioned in 4 cases. Infants under 1 year of age constituted 13/19 (68.4%) of the cohort. The remaining cases included 3 adolescents, 1 adult, and 2 patients with unreported age. At the onset of gray baby syndrome symptoms, the cumulative chloramphenicol dose ranged from 95 mg/kg to 4,900 mg/kg. Serum chloramphenicol levels in these patients ranged from 30 μg/mL to 313 μg/mL. Notably, serum chloramphenicol levels exceeded 50 μg/mL in all patients except one (30 μg/mL). Herein, 11 of 17 cases with reported outcomes survived (64.7%), whereas the remaining 6 died (35.3%). The clinical manifestations observed in the present case, including vomiting, lethargy, pallor, impaired peripheral perfusion, respiratory distress, metabolic acidosis, reduced cardiac ejection fraction, ventricular dilation, and hepatomegaly, are consistent with the classic presentation of gray baby syndrome described in previous reports.

**Table 2:** Cases of gray baby syndrome reported.

Reference source	Location (city/pro vince)	Age	Sex	Weight (kg)	Comorbidities	Indication for CAP	CAP dosage	Onset time from initiati on	Cumulati ve dose at onset	Serum level at onset (μg/m L) Not mentioned	Clinical manifestations	Interventions	Outcome
Morton (17) 1961	Kennewi ck	6 weeks	Female	4.53	No	Bronchopneumonia	55 mg/kg/dose, q6 h, IM	12 h	166 mg/kg	98	Abdominal distension, cyanosis, hypotension, irregular breathing, apnea, and oliguria	Endotracheal intubation, nasogastric tube placement, and fluid resuscitation Charcoal-column hemoperfusion, hemodialysis, protamine sulfate, and sodium bicarbonate	Survived*
Mauer et al. (10) 1980	Minneapolis	12 days	Male	3.2	Bilateral nephrostomy tube placement and continuous urethral catheter drainage	Suspected sepsis	Administered at 50 mg/kg/dose, q6 h, IV due to a dosing error, intended dose: 50 mg/kg/day. The initial dose of CAP was erroneously administered at 250 mg/kg, IV, due to a dosing calculation error, intended dose: 25 mg/kg/day	42 h	350 mg/kg	135	Ashen-gray skin discoloration, cyanosis, mottling, hypotension, acidosis, lethargy, and tachypnea	Endotracheal intubation, serial exchange transfusions, dopamine infusion, and blood transfusion	Recovered
Kessler et al. (18) 1980	Seattle	85 h	Female	3.4	No	The CSF and urine cultures grew *Escherichia coli* that were resistant to ampicillin but sensitive to gentamicin and chloramphenicol. *Haemophilus influenzae* meningitis	D1-D2: 100 mg/kg/day, IV; D8: 100 mg/kg/day, q4 h, IV; D9: A single dose of 1,100 mg was administered due to a dosing calculation error, intended single dose: 110mg	16 h	250 mg/kg	180	Ashen-gray skin discoloration, metabolic acidosis, hepatic impairment with hepatomegaly, hypotension, oliguria, and two episodes of cardiopulmonary arrest	Mechanical ventilation and three serial exchange transfusions	Recovered
Stevens et al. (19) 1981	Indianapolis	5 weeks	Male	4.6	No	Rocky Mountain spotted fever	D1: 23.8 mg/kg/dose, q6 h IV; D2: 22.3 mg/kg/dose, q6 h, IV; D3: 23.8 mg/kg/dose, q6 h, PO	6 h	539 mg/kg	140	Lethargy, pallor, hypothermia, and recurrent apnea	Fluid replacement and mannitol	Death
Brown (14) 1982	Birmingh am	16 years	Female	42.1 5	No	Suspected meningitis	Administered at 75 mg/kg/dose four times daily due to a calculation error, intended dose: 75 mg/kg/day	1 D	95 mg/kg	136	Agitation, vomiting, hypotension, acidosis, rapid breathing, and sluggish pupillary reflex	Endotracheal intubation, charcoal-column hemoperfusion, sodium bicarbonate, multiple vasoactive agents, and exchange transfusion	Recovered
Freundlich et al. (8) 1983	Miami	7 weeks	Male	4.6	No	Ampicillin-resistant	50 mg/kg/day, IV	54 h	675 mg/kg	277	Lethargy, rapid breathing, decreased muscle tone, pale/grayish skin, unresponsive to stimuli, peripheral circulatory failure, hypotension, and cardiopulmonary arrest	Maximal resuscitative efforts attempted (specifics undocumented)	Death
Fripp et al. (13) 1983	Hershey	3 months	Not mentioned male	Not mentioned	No	*Haemophilus influenzae* type B meningitis	100 mg/kg/day, IV	8 D	350 mg/kg	84	Agitation, vomiting, hypotension, and ventricular enlargement	Endotracheal intubation, cardiopulmonary resuscitation (CPR), epinephrine, sodium bicarbonate, and calcium carbonate	Death
Evans and Kleiman (11) 1986	Indianapolis	4 months	Not mentioned	Not mentioned	Bronchitis	Suspected sepsis	100 mg/kg/day	41 h	Not mentioned	62	Abdominal distension, acidosis, hypotension, and hypothermia	Not mentioned	Survived with profound psychomot or retardation
Evans and Kleiman (11) 1986	Indianapolis	Not mentio ned	Not mentioned	Not mention	Hypoaldosteronism	Suspected sepsis	100 mg/kg/day	40 h	Not mentioned	80	Acidosis, hypotension, and hypothermia	Not mentioned	Survived*
Evans and Kleiman (11) 1986	Indianapolis	Not mentio ned 11 years	Not mentioned male	No	Dysautonomia	Suspected sepsis	75 mg/kg/day	40 h	Not mentioned	30	Abdominal distension, acidosis, hypotension, and hypothermia	Not mentioned	Survived*
Evans and Kleiman (11) 1986	Indianapolis	3.5 months			Reye syndrome	Suspected sepsis	75 mg/kg/day	81 h	Not mentioned	75	Abdominal distension, acidosis, hypotension, and hypothermia	Emergency aortic valve replacement surgery	Survived*
Spear and Wetzel (20) 1987	Baltimore				Pneumococcal meningitis	Suspected bacterial endocarditis (16)		48 h	150 mg/kg		Hypotension, hypothermia, acidosis, ventricular enlargement, and decline in cardiac systolic function		Death
Spear and Wetzel (20) 1987	Baltimore	3 weeks	Male	No	Transposition of the great arteries, coarctation of the aorta, ventricular septal defect, atrial septal defect and patent ductus arteriosus, and congestive heart failure	Postoperative prophylaxis against potential infection following repair of coarctation of the aorta and ligation of patent ductus arteriosus/pulmonary artery	70 mg/kg/day	96 h	280 mg/kg	70	Hypotension, hypothermia, acidosis, pale/grayish skin, oliguria, cardiac chamber enlargement, decline in cardiac systolic function, and hepatic impairment	Endotracheal intubation, dopamine, dobutamine, isoproterenol, and sodium bicarbonate	Survived*
Zhao (21) 1990	Shanxi Province	11 h	Male	Not mention ed	Cellulitis of the right thigh	Neonatal sepsis	100 mg/day, IV	5 days	500 mg	Not mentio ned	Abdominal distension, vomiting, feeding refusal, ascites, grayish complexion, irregular breathing, apnea, and poor peripheral perfusion	Volume expansion and acidosis correction	Discharge against medical advice*
Suarez and Ow (22) 1992	Maywoo d	9 months	Male	Not mention ed	Facial cellulitis	Suspected sepsis	75 mg/kg/day	5 days	300 mg/kg	313	Lethargy, cyanosis, hypothermia, hypotension, tachypnea, cardiomegaly, and tachycardia	Endotracheal intubation, digoxin, dopamine, furosemide, blood transfusion, and blood products	Recovered
Wang and Chen (16) 2003	Jilin Province	22 years	Female	Not mentioned	No	Self-administered	270 tablets (specification unknown), PO	5 h	270 tablets (specificat ion unknown)	Not mentioned	Dizziness, dyspnea, hypotension, shock, bilaterally dilated pupils with sluggish light reflex, decreased limb muscle tone, and pulmonary edema	Endotracheal intubation, Peritoneal dialysis, dopamine, dobutamine, naloxone, norepinephrine, metaraminol, Cedilanid, sodium bicarbonate, methylprednisolone, and ranitidine	Death
Wang (23) 2005	Jinan	3 months	Female	Not mentioned	No	Mother exclusively breastfeeding while taking chloramphenicol, consequently passively administering chloramphenicol to the infant via breast milk, and at the same time, she administered chloramphenicol to the infant.	Not mentioned	Not mentioned	Not mentioned	Not mentioned	Vomiting, diarrhea, feeding refusal, irregular breathing, and circulatory failure	Not mentioned	Not mentioned*
Wiest et al. (15) 2012	Charlesto n	12 years	Male	45	Allergy to penicillin and ceftriaxone; childhood history of asthma and febrile seizures.	Brain abscess	22 mg/kg/dose, q6 h, IV	50 days	4.9 g/kg	61	Abdominal distension, lethargy, and acidosis	Hemodialysis, vitamin B_12_ and vitamin B₆	Survived with visual field deficits as permanent sequelae
Liu et al. (24) 2015	Yunnan Province	1 years	Male	9	No	Accidental ingestion	1,000 mg, PO	4.5 h	111 mg/kg	Not mentioned	Vomiting, abdominal distension, hypotension, agitation, lethargy, cyanosis, dyspnea, atrial fibrillation, acidosis	Gastric lavage, urinary catheterization, endotracheal intubation, cardiopulmonary resuscitation, epinephrine, methylprednisolone, deslanoside, mannitol, sodium bicarbonate, furosemide, vitamin C, vitamin B₆, ceftriaxone, and cimetidine	Death

The asterisk (*) in Table 2 denotes missing follow-up information after discharge.

CAP, chloramphenicol; CSF, cerebrospinal fluid.

Among the 19 reported gray baby syndrome cases, 15 occurred in the United States and 4 in China. The US cases were predominantly concentrated within the first four decades since chloramphenicol's introduction into the market (13/15, 87%), with most of these cases reported from relatively economically developed urban areas. This geographic pattern may be attributable to greater accessibility to chloramphenicol in affluent regions at that time. Reports of chloramphenicol-induced gray baby syndrome have subsequently declined, coinciding with the introduction and widespread adoption of newer antibiotics. Conversely, all Chinese cases were reported after the year 2000 and originated exclusively from non- tier- one cities. In China, the Guidelines for Clinical Use of Antimicrobial Agents issued by the National Health Commission (formerly the Ministry of Health) in 2004 and 2015 both state that the clinical use of chloramphenicol has declined significantly due to increasing bacterial resistance and serious adverse effects such as bone marrow suppression. Nevertheless, chloramphenicol retains specific clinical indications owing to its excellent tissue penetration—including across the blood- brain and blood- ocular barriers—and its efficacy against intracellular pathogens such as Salmonella typhi and Rickettsia. The guidelines emphasize the necessity of regular blood- count monitoring during treatment. Chloramphenicol is contraindicated in premature and newborn infants because of the risk of gray baby syndrome, and therapeutic drug monitoring is required when its use in infants and young children is unavoidable (25, 26). However, in certain less- developed regions of China, inappropriate use of chloramphenicol in specific populations remains a concern due to its low cost and easy accessibility, which increases the risk of accidental ingestion by children or inappropriate prescribing by clinicians. Therefore, it must be strongly emphasized that chloramphenicol should be strictly avoided in children unless a clear clinical indication exists.

In addition, chloramphenicol use requires particular caution in children with severe malnutrition, hepatic impairment, or underlying cardiac disease as impaired drug clearance in these populations increases the risk of toxicity. In such high-risk patients, close monitoring of serum drug concentrations and vigilant assessment for signs of toxicity are essential (20, 27). For parents, ensuring that chloramphenicol is properly stored and kept out of reach is essential to prevent accidental ingestion by young children.

For patients suspected as having gray baby syndrome, serum chloramphenicol levels should be monitored if feasible. Imaging studies should encompass echocardiography, and electrocardiogram (ECG) is also indicated. Management of gray baby syndrome focuses on increasing chloramphenicol elimination and providing supportive care. Prompt removal of unabsorbed drug may involve induction of emesis, gastric lavage, or administration of cathartics. For absorbed drug clearance, aggressive IV fluid resuscitation and diuresis promote excretion; extracorporeal purification (e.g., CRRT and charcoal hemoperfusion) is considered for severe cases. Hemodynamic instability requires vigorous fluid resuscitation, supplemented with inotropes/vasopressors in cases of circulatory failure. Respiratory support (oxygen and mechanical ventilation) is provided as needed. Maintaining homeostasis involves correcting metabolic acidosis and electrolyte imbalances. Rewarming should be performed as needed, and hypoglycemia should be promptly corrected. Hepatoprotective agents (e.g., glucurolactone and glutathione) are commonly used to facilitate recovery from liver injury, and prophylactic B vitamins (B1, B12, and folate) are typically given to mitigate aplastic anemia risk (24). In the present case, gastric lavage, endotracheal intubation, CRRT, fluid resuscitation, acidosis correction, vasoactive agents, hepatoprotective therapy, cardioprotective measures, and stress ulcer prophylaxis aligned with management strategies documented in the existing literature and yielded favorable outcomes, including complete restoration of hepatic, renal, and cardiac function.

## Data Availability

The original contributions presented in the study are included in the article/Supplementary Material, further inquiries can be directed to the corresponding author.
